# Orai3 Constitutes a Native Store-Operated Calcium Entry That Regulates Non Small Cell Lung Adenocarcinoma Cell Proliferation

**DOI:** 10.1371/journal.pone.0072889

**Published:** 2013-09-13

**Authors:** Anne-Sophie Ay, Nazim Benzerdjerb, Henri Sevestre, Ahmed Ahidouch, Halima Ouadid-Ahidouch

**Affiliations:** 1 Laboratory of Cellular and Molecular Physiology, LPCM: EA 4667, SFR CAP-SANTE (FED 4231), UFR of Sciences, Amiens, France; 2 Pathological Anatomy and Cytology Service, Amiens North Hospital, Amiens, France; 3 Department of Biology, Ibn Zohr University, Agadir, Morocco; Thomas Jefferson University, United States of America

## Abstract

Orai channels have been associated with cell proliferation, survival and metastasis in several cancers. The present study investigates the expression and the role of Orai3 in cell proliferation in non-small cell lung cancer (NSCLC). We show that Orai3 is over-expressed in cancer tissues as compared to the non-tumoral ones. Furthermore, Orai3 staining is stronger in high grade tumors. Pharmacological inhibition or knockdown of Orai3 significantly reduced store operated calcium entry (SOCE), inhibited cell proliferation and arrested cells of two NSCLC cell lines in G0/G1 phase. These effects were concomitant with a down-regulation of cyclin D1, cyclin E, CDK4 and CDK2 expression. Moreover, Orai3 silencing decreased Akt phosphorylation levels. In conclusion, Orai3 constitutes a native SOCE pathway in NSCLC that controls cell proliferation and cell cycle progression likely via Akt pathway.

## Introduction

Calcium is a key messenger that regulates proliferation, apoptosis, migration and invasion, which are the main mechanisms implicated in cancer progression [1], [2]. It controls G1 progression, G1/S and G2/M transition phases by regulating expression of several calcium-dependent signaling pathways, such as calmodulin, CaM-Kinase, and calcineurin [3], [4], [5]. Therefore, identification of the pathways involved in Ca^2+^ influx regulating the cell proliferation are in the focus of a number of investigations.

In epithelial cells, store-operated Ca^2+^ entry (SOCE) is the main route that drives most Ca^2+^-dependent signaling cascades [6], [7], [8]. Recent studies have reported that Orai1 and Stim1 regulate store-operated calcium influx [9], [10], [11], cell proliferation [12], [13] and cell cycle progression [13], [14], [15]. However, studies of principal components of store-operated Ca^2+^ entry in lung cancer cells are limited. Indeed, only three recent studies have shown the involvement of Orai1 and TRPC1 in SOCE in lung cancer A-549 cell line [16], [17]. Over-expression of Orai1 or down-regulation of TRPC1 decreases the expression of cyclin D (D1 and D3), arrests cells at G1 phase and inhibits the EGF proliferative effect [16], [17]. Furthermore, Inhibition of Orai1 reduces the NFκB activation.

It has been demonstrated recently that Orai3 is a part of a native store-operated Ca^2+^ entry pathway in the estrogen receptor (ER) positive breast cancer cells [18]. We have previously reported that down-regulation of Orai3 inhibits cancer cell proliferation, contributes to cell cycle arrest in G1 phase, and increases apoptotic cell death [19]. Interestingly, Orai3 silencing does not affect cell proliferation in the non tumorigenic MCF-10A cells [19]. Given the above we propose that Orai3 may be of crucial significance in the human lung cancer where altered calcium homeostasis could favor cell growth. Therefore, the objectives of our study were to determine Orai3 expression in lung cancer tissue samples and to establish its role in cell proliferation and survival using two NSCLC cell lines representing the most common malignant lung tumor. We demonstrate that Orai3 expression is up-regulated in lung cancer tissues, correlates with high tumor grade, and Orai3-mediated Ca^2+^ entry is crucial to NSCLC cell proliferation.

## Materials and Methods

### Patients: ethical agreement

Ethical approval for this study was granted by the “Comité Consultatif de Protection des Personnes dans la Recherche Biomédical de Picardie” (2012/43- N° ID-RCB: 2012-A01542-41-CANIO-Poumon). The study has been performed on 60 healthy and cancer samples obtained from patients with lung adenocarcinoma received a surgical resection, between 2005 and 2011 at the University Hospital of Amiens. Lung tissue samples were collected from fully informed patients provided written consent prior to clinical procedures. Several histological factors representing important prognostic factors for lung adenocarcinoma were measured, e.g. tumor grade of differentiation (1 = well-, 2 = moderately-, and 3 = poorly differentiated), vascular invasion, and necrosis. The other parameters of the epidemiological study are: population characteristics (age, sex), tumor size, lymph node metastasis (number of lymph nodes examined), tumor, and death.

### Immunohistochemistry

Immunohistochemistry was performed on 60 tissue samples of lung adenocarcinoma. Briefly, four-micrometer-thick sections of formalin-fixed and paraffin-embedded tissue samples were sliced from the tissue block. Immunohistochemical staining was performed on a Roche Ultra immunostainer, using antibody directed against Orai3 (rabbit polyclonal, 1∶100 dilution, Sigma, Saint Louis, USA). This was followed by the avidin-biotin-peroxidase complex technique. Reactions were developed using a chromogenic reaction in DAB (diamino-3,3′benzidine tetrahydrochloride) substrate solution (DAB, Sigma Fast). Counterstaining was carried out with hematoxylin solution. This antibody is certified for immunohistochemistry by Sigma Inc. A negative control was performed using the same technique without the primary antibody.

Immunostaining levels for Orai3 were determined by subjective visual scoring of the brown stain. Two operators independently evaluated antigen expression.

Because of a heterogeneous staining of the tumor cells, we chose a score of staining that matched level of intensity (0, 1, 2 or 3) multiplied by proportion of cell staining.

### Cell culture

NCI-H23 and NCI-H460 cells were grown in Eagle's Minimum Essential Medium (EMEM) supplemented with 10% fetal calf serum (Lonza, Levallois-Perret, France), 2 mM L-glutamine, and 0.06% HEPES. Both cell lines were grown in a 5% CO_2_-humidified incubator at 37°C.

### Real-time quantitative PCR

Total RNA from cell lines were extracted as previously described [19]. For the PCR reaction, sense and anti-sense PCR primers specific to Orai1 (for 5′- AGGTGATGAGCCTCAACGAG-3′,rev 5′-CTGATCATGAGCGCAAACAG-3′), Orai2 (for 5′- GCAGCTACCTGGAACTGGTC-3′ and rev 5′- CGGGTACTGGTACTGCGTCT-3′), Orai3 (for 5′-AAGTCAAAGCTTCCAGCCGC-3′; and rev 5′-GGTGGGTACTCGTGGTCACTCT-3′) and ß-actin (for 5′-CAGAGCAAGAGAGGCATCCCT-3′; and rev 5′-ACGTACATGGCTGGGGTG-3′) were used. Real-time PCR was performed as previously described [19]. The relative amount of Orai1, Orai2 or Orai3 “target” was normalized to the endogenous control (β-actin) and compared to the reference sample (lung cells transfected with si-CTL) using the Pfaffl method.

### Cell-transfection

Transfection of cells was performed using the nucleofection technology according to the Amaxa Biosystems protocol. Cells were transfected with 4 µg of siRNA directed against Orai1 (5′-GCC AUA AGA CUG ACC GAC A -3′) or Orai2 (5′-GCC ACA ACC GUG AGA UCG A-3′) or Orai3 [19], with scrambled siRNA as a control (siGENOME Non-Targeting siRNA, Dharmacon Research Inc., Chicago, IL, USA) in V kit solution and X-013 program (Amaxa Biosystems).

### Cell viability and mortality

Cell viability and mortality were assessed by Trypan Blue assay. After transfection with si-Orai3, or with functional non-coding si-CTL as previously described, NCI-H23 and NCI-H460 were grown in 35-mm Petri dishes at a density of 20×10^4^ cells for 72- h. Cell viability and mortality was assessed using the standard Malassez cell method [19].

### Cell cycle analysis

To measure the cellular DNA content, adherent cells transfected by si-Orai3 or si-CTL for 72-h were collected by trypsinization. Cells were pelleted, resuspended in PBS/EDTA, treated with 20 µg/ml RNase A (Sigma-Aldrich), and stained with 50 µg/ml of Propidium Iodide (Sigma-Aldrich). Samples were analyzed on BD Accuri C6 flow cytometer. The percentage of cells in different phases was calculated using FlowJo software (Tree Star, Inc).

### Western blot analysis

Whole-cell lysates were prepared with 1% sodium dodecyl sulphate and a protease inhibitor cocktail (Sigma-Aldrich, France). Proteins were separated by denaturing SDS-PAGE and transferred onto nitrocellulose membranes. The primary antibodies used were: anti-CDK2 (1∶2000, Santa Cruz Biotechnology, Inc., Heidelberg, Germany), anti-CDK4 (1∶1200, Santa Cruz Biotechnology, Inc., Heidelberg, Germany), anti-cyclin E (1∶1200, Santa Cruz Biotechnology, Inc., Heidelberg, Germany), anti-cyclin D1 (1∶500, Cell Signalling Tech., Danvers, MA, USA), anti-Orai3 (1∶200, Sigma, Poway, CA, USA), anti-Orai1 (1∶200, Sigma, Poway, CA, USA), anti-P-Akt (1∶250, Cell Signalling Tech., Danvers, MA, USA), anti-Akt (1∶750, Cell Signalling Tech., Danvers, MA, USA). Antibodies are followed by secondary antibodies coupled to horseradish peroxidase. β-actin antibody (1∶1000, Santa Cruz Biotechnology, Inc., Heidelberg, Germany) was used for loading control experiments. Bands were detected using an enhanced chemiluminescence kit (GE Healthcare, Saclay, France) and quantified using the densitometric analysis option in the Bio-Rad image acquisition system (Bio-Rad Laboratories, France).

### Apoptosis analysis

To evaluate the percentage of apoptotic cells, we measured cell surface exposure of phosphatidylserines by FITC Annexin V Apoptosis Detection Kit I (BD Biosciences Pharmingen, France). Both detached and adherent cells were collected, washed twice in ice-cold PBS and re-suspended in 1× binding buffer. After staining, the samples were analyzed with BD Accuri C6 flow cytometer.

### Ca^2+^ measurements

Cells were plated onto glass coverslips after transfection with si-CTL or si-Orai3 or without transfection. The cytosolic calcium concentration was measured using FURA-2 loaded cells. Cells were loaded for 1 h at 37°C with 3 µM of Fura-2-AM prepared in culture medium. Before recording, cells were washed twice with the extracellular dye free solution (NaCl 140 mM, KCl 5 mM, CaCl_2_ 2 mM, MgCl_2_ 2 mM, D-glucose 5 mM, and HEPES 10 mM, pH 7.4). The glass coverslip was mounted in a chamber on a Zeiss microscope equipped for fluorescence. Fura-2 fluorescence was excited to 350 and 380 nm using a monochromator (Polychrome IV; TILL Photonics, Planegg, Germany), and fluorescence captured by a Cool SNAPHQ camera (Princeton Instruments, Evry, France) after filtration through a long-pass filter (510 nm). Metafluor software (version 7.1.7.0) was used for acquisition and analysis. All recordings were made at room temperature. The cells were continuously perfused with a saline solution and chemicals were added as indicated. The flow rate of the whole cell chamber perfusion system was set to 1 ml/min with the chamber volume of 500 µl.

### Statistical analysis

Data are presented as Mean ± S.E.M., “n” refers to the number of experiments. Statistical analyses were performed using Mann–Whitney or paired t-tests, as appropriate using Sigma-Stat 3.0 (Systat Software, Inc.). When more than two conditions were compared, a Kruskal-Wallis one way ANOVA was employed followed by Dunn's Method *post-hoc* tests using SigmaStat 3.0 (Systat Software, Inc.). Differences were considered significant at *p*<0.05.

## Results

### Orai3 is overexpressed in lung adenocarcinoma and correlated with high tumor grade

**Figure 1:**
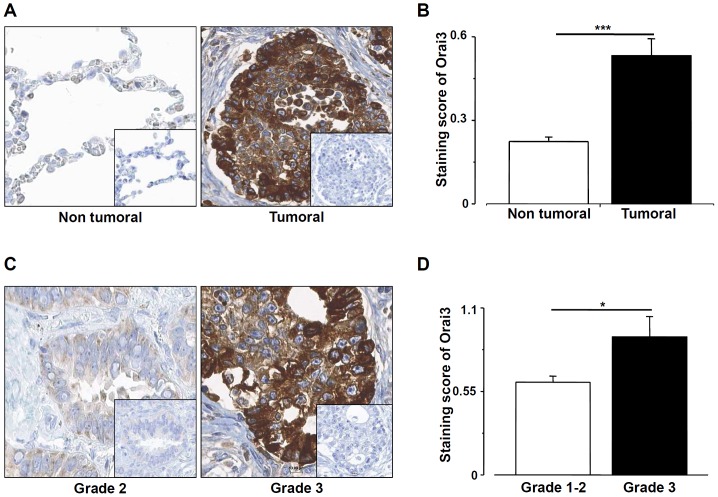
Orai3 is overexpressed in lung adenocarcinoma and correlated with high tumor grade. A: Representative examples of Orai3 expression in cancerous and matched non-tumoral human lung tissues, as assessed by immunohistochemistry. Original magnification: ×400. *Insert* negative control obtained by omitting the primary antibody. B, quantitative analysis of Orai3 staining score in tumoral and adjacent non-tumoral lung tissue (n = 60, *** *p*<0.001, Mann-Withney test). C, Typical examples of Orai3 immunoreactivity in grade 2 lung cancer tissue (weak staining) and in grade 3 lung adenocarcinoma tissue (strong staining) at a magnification of ×400. *Inserts* negative control obtained by omitting the primary antibody. D, quantification of Orai3 staining score in grade 1–2 versus grade 3 lung cancer (n = 40, *p* = 0.032, Mann-Withney test).

The expression of Orai3 was analyzed by immunohistochemistry in 60 tumors and adjacent non-tumoral tissue samples obtained from the same patient. A strong staining of Orai3 was observed in 66.7% of tumor samples as compared to the non-tumoral samples (40/60, *p*<0.001). A representative Orai3 staining is shown in Figure 1A. Quantitative analysis of the Orai3 expression revealed that Orai3 staining score significantly increased in tumors (0.53±0.06, n = 40) as compared to non tumoral epithelial tissue from the same patient (0.22±0.01, n = 20, *p*<0.001, Fig. 1B).

The Orai3 staining score was elevated in higher tumor grade (grade 3; 0.92±0.13; n = 16) as compared to low tumor grades (grade 1–2; 0.61±0.04; n = 24, *p* = 0.032, Fig. 1C–D). We also assessed correlations of the Orai3 expression with clinical parameters (age, sex, tumor size, nodal metastasis, vessel invasion, TNM). No correlations were found between Orai3 expression and clinical parameters (see Table 1). Altogether, these results provide evidence that Orai3 is overexpressed in lung adenocarcinoma and the level of its expression correlates with high tumor grade.

**Table 1:** Clinical characteristics of patients who underwent pulmonary resection for adenocarcinoma.

Characteristic	Orai3>0.3	Orai3<0.3	P-value
Total participants	40	20	
Age (y), mean±SD	62.9±10.8	58.2±9.3	0.12
Sexe			
Male	28	16	0.54
Female	12	4	
Tumor size (y), mean±SD			0.13
≤30 mm	32	10	0.06
>30 mm	8	10	0.368
Nodal metastasis			0.28
N0	32	12	0.13
N1	3	5	0.13
N2	5	3	0.67
Vessel invasion			
Positive	6	5	0.87
Negative	34	15	
P stage			
I	25	12	0.62
II	7	4	0.67
III	5	3	0.67
IV	3	1	0.89

### NSCLC cells possess store operated calcium entry (SOCE) and express functional Orai3

**Figure 2:**
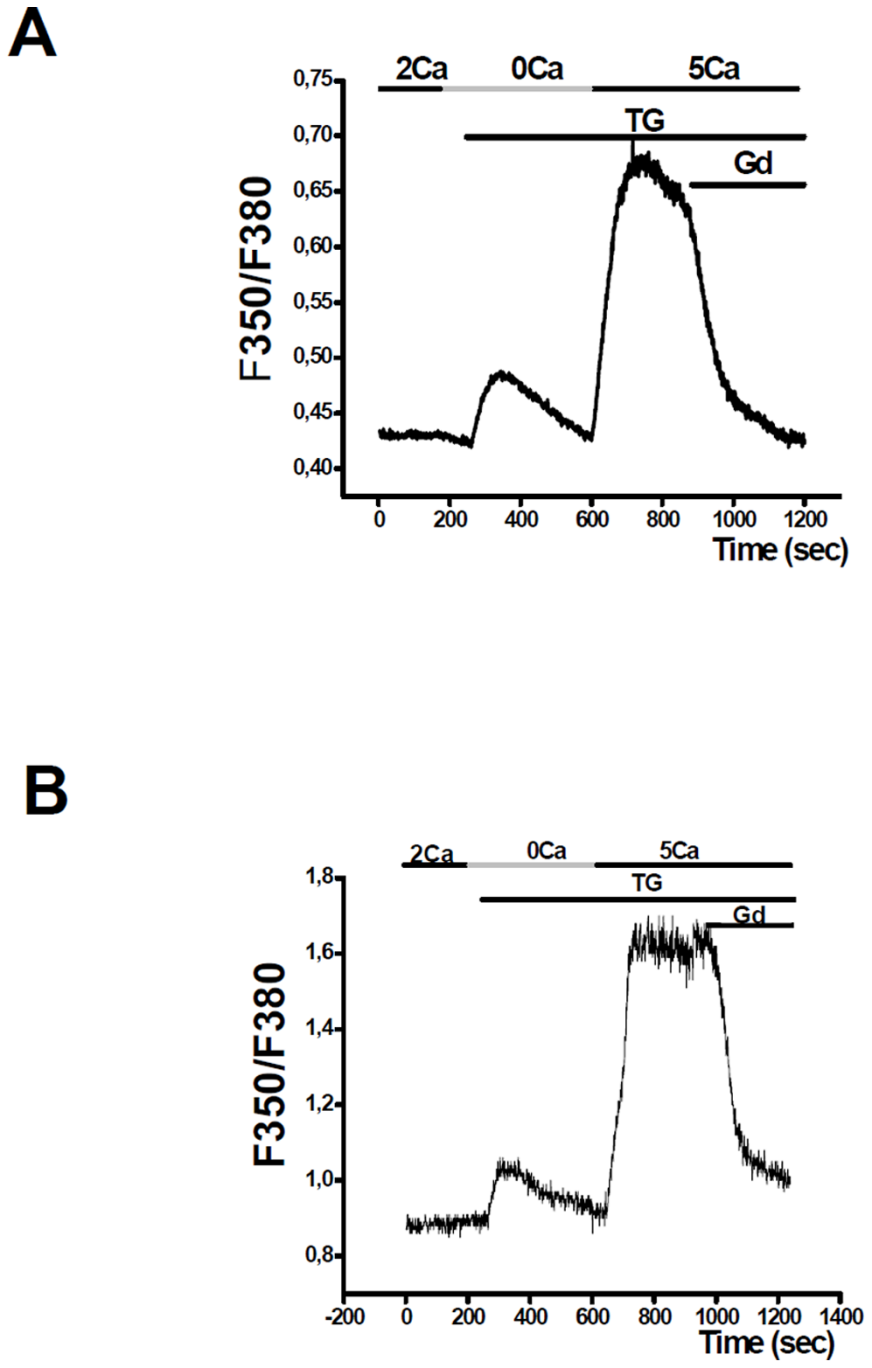
NSCLC cells display SOCE. Thapsigargin (TG, 1 µM) applied to NCI-H23 cells (A) or NCI-H460 (B) in 0 mM Ca^2+^ solution (0Ca) induced an increase of F350/F380 fluorescence. Re-introduction of 5 mM Ca^2+^ solution (5Ca) induced stable increase in Ca^2+^ signal, a characteristic of SOCE. Gd^3+^ applied in 5Ca solution induced inhibition of this Ca^2+^ signal. (*p*<0.001, n = 25 for NCI-H23 and n = 28 for NCI-H460, Mann-Withney test).

We characterized SOCE in two NSCLC cell lines: NCI-H23 and NCI-H460. We used a pharmacological inhibitor, Gd^3+^ known to inhibit at low concentration native SOCE in many cell types [20], [21]. Intracellular Ca^2+^ concentration was monitored by ratiometric imaging in the cells loaded with Fura-2. SOCE was monitored through thapsigargin-stimulated Ca^2+^ influx. We first perfused 1 µM thapsigargin in Ca^2+^ free solution for 7 min. Then, 5 mM Ca^2+^ was added. The perfusion of 5 µM Gd^3+^ almost completely suppressed SOCE in both cell lines (Fig. 2A–B, *p*<0.001, n = 25 for NCI-H23 and n = 28 for NCI-H460).

**Figure 3:**
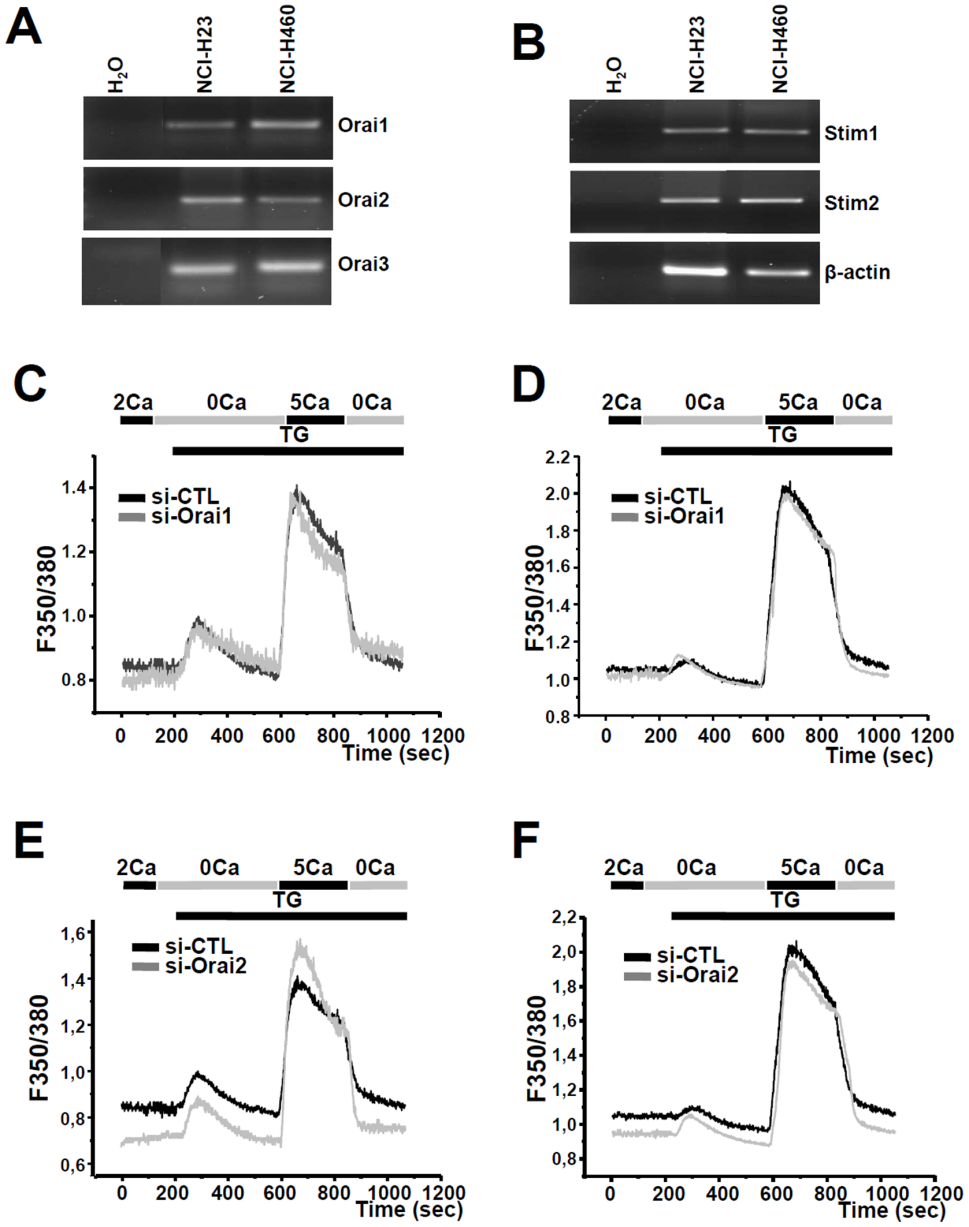
Silencing of Orai1 and Orai2 does not change SOC entry in the two cell lines. Expression of Orai1, Orai2, Orai3, Stim1, and Stim2 estimated in NCI-H23 (A) and NCI-H460 (B) cells using semi-quantitative RT-PCR. si-Orai1 has no effect on SOCE in NCI-H23 (C, n = 125) and NCI-H460 (D, n = 150). Silencing Orai2 (si-Orai2) induces increase in SOCE in NCI-H23 (E, n = 125, p<0.001, Mann-Withney test), but has no effect on SOCE in NCI-H460 (F, n = 125) cells.

We next examined the Orai expression in lung adenocarcinoma cell lines: NCI-H23 and NCI-H460 cell lines using RT-PCR. As shown in Figure 3A–B, both NCI-H23 and NCI-H460 cells express Orai1, Orai2, Orai3, Stim1 and Stim2.

Using Fura2 Ca^2+^ imaging, we show that knockdown of Orai1 had no significant effect on SOCE in NCI-H23 (Fig. 3C, 3% decrease, n = 125,) and NCI-H460 cells (Fig. 3D, n = 150). However, silencing of Orai2 induced an increase in SOCE in NCI-H23 (35%, n = 125, p<0.001, Fig. 3E) and did not change SOCE in NCI-H460 cells (n = 125, Fig. 3F). The efficiency of the siRNA sequences (si-Orai1, si-Orai2) was confirmed with mRNA measurements in NCI-H23 and NCI-H460. A 72-h treatment with these siRNA significantly reduced mRNA of Orai1 and Orai2 by 41.35±10.6% and 59.74±12.5%, respectively, in NCI-H23 (Fig. S1A) and by 33.88±5.8% and 57.07±12.4%, respectively, in NCI-H460 (Fig. S1B).

**Figure 4:**
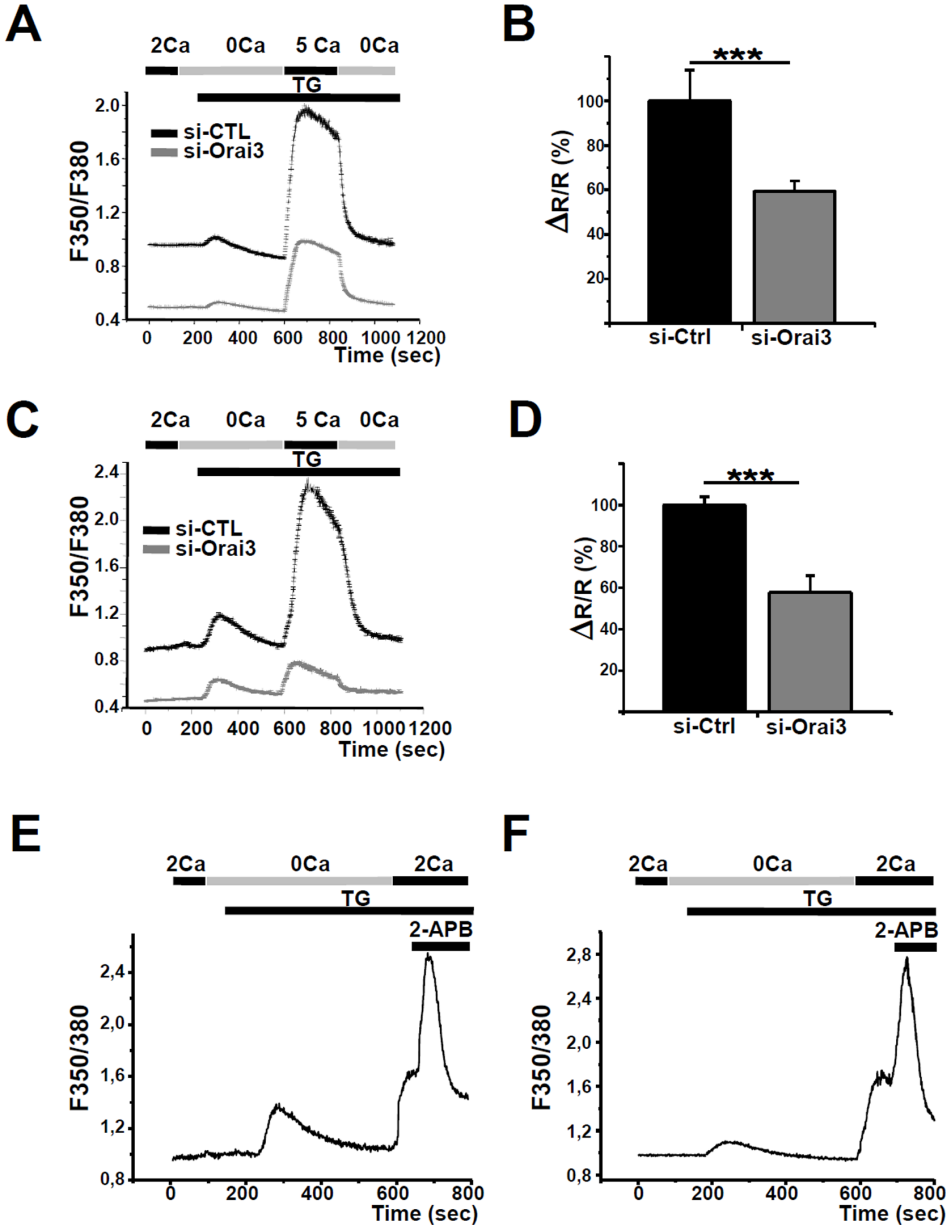
Effect of Orai3 knockdown on SOC entry. The effect of siRNA against Orai3 on thapsigargin-activated SOCE in (A) NCI-H23 cells (n = 25 cells for each condition) and (C) NCI-H460 cells (n = 27 cells for each condition). Down-regulation of Orai3 expression produced a significant reduction of basal calcium concentration. [Ca^2+^]_i_ is measured after 1 minute of standard solution extracellular perfusion. B–D) Quantitative analysis of SOCE in NCI-H23 cells (B, n = 25) and NCI-H460 cells (D, n = 27). The values presented as mean ± SEM. (***p<0.001, Mann-Withney test). Perfusion of 50 µM 2-APB transiently potentiates SOCE in NCI-H23 (E, n = 45) and NCI-H460 cells (F, n = 54). The values presented as mean ± SEM. (***p<0.001, Mann-Withney test).

To provide direct evidence for the contribution of Orai3 in SOCE, we silenced Orai3 using specific siRNA. Both Orai3 transcripts and protein levels were decreased in NCI-H23 and NCI-H460 cells transfected with the siRNA (Fig. S2A–B). Orai3 silencing failed to affect Orai1 at both transcripts and proteins levels in both cell lines (Fig. S2C–D). Silencing of Orai3 significantly reduced both basal intracellular Ca^2+^ concentration ([Ca^2+^]i) (16±7%, n = 25, for NCI-H23 cells and 32±3%, n = 27, for NCI-H460 cells, *p*<0.001), and thapsigargin-activated SOCE in NCI-H23 (41±9% (n = 25), *p*<0.001, Fig. 4A–B) and NCI-H460 cells (43±4%, n = 27, *p*<0.001, Fig. 3C–D). To investigate contribution of Orai3 in the Ca^2+^ entry, we analyzed Mn^2+^ quenching of fura-2 fluorescence. Addition of Mn^2+^ decreased the fura-2 fluorescence in a time-dependent manner revealing a Mn^2+^ influx though plasma membrane Ca^2+^ channels [22]. Upon removal of the culture medium supplemented with 10% serum prior to experimental acquisition, Orai3 silencing drastically decreased Mn^2+^ quenching in both cell lines cells (Fig. S3A–B). This result suggested that Orai3 is activated under culture conditions. To strengthen Orai3 involvement in SOCE, we used 2-APB, which activates Orai3 but inhibits Orai1 and Orai2 [23], [18]. Perfusion of 50 µM 2-APB induced a transient and robust increase of SOCE in NCI-H23 (Fig. 4E) and NCI-H460 (Fig. 4F). Thus, our pharmacological and siRNA data indicate that Orai3 is the major SOCE channel in these two cell lines.

### Down-regulation of Orai3 reduces cell proliferation and induces cell arrest in G1 phase of the cell cycle

**Figure 5:**
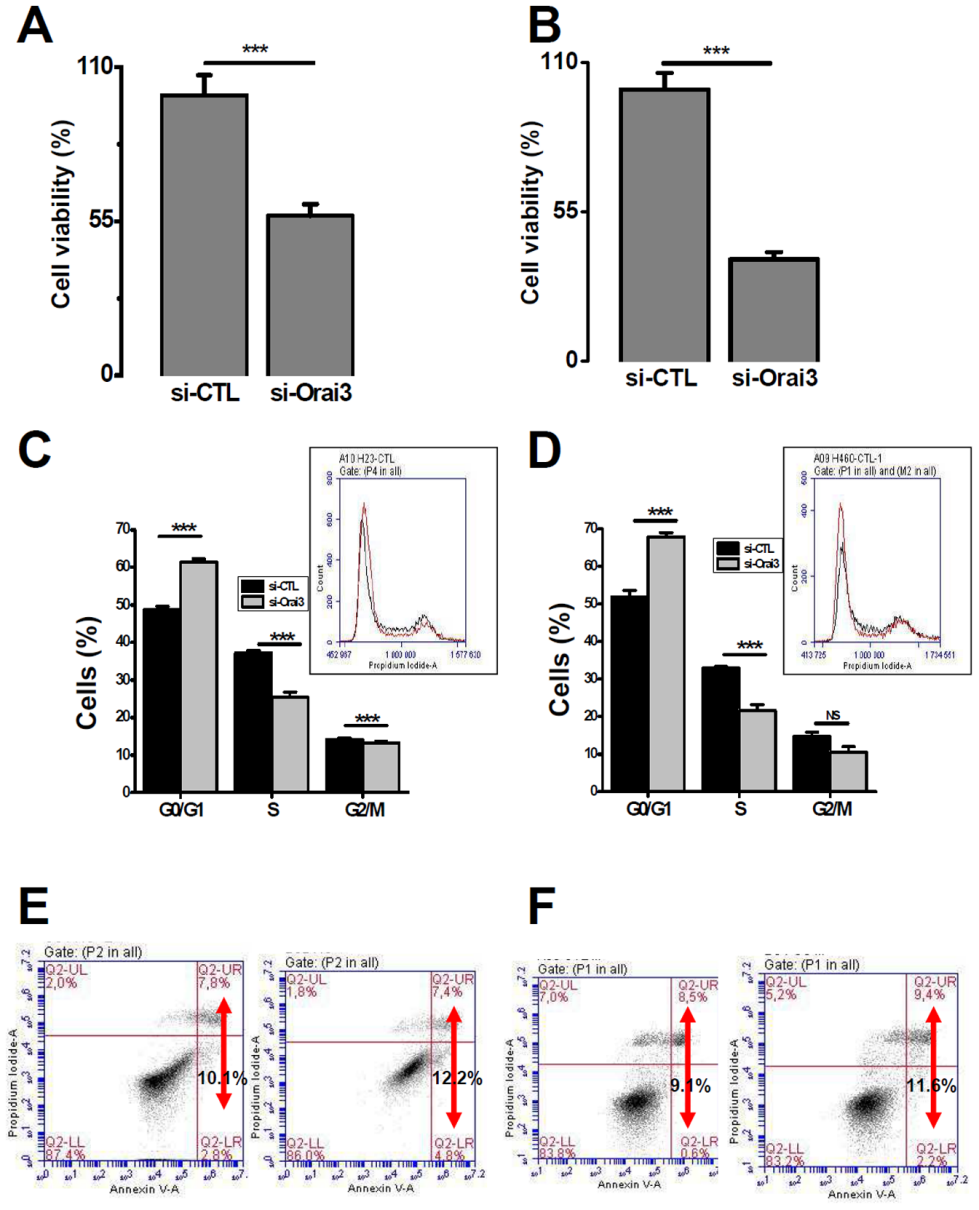
Down regulation of Orai3 reduced cell proliferation and arrested cells in G0/G1 phase. A, Effect of Orai3-knockdown on NCI-H23 cell viability (*** *p*<0.001, *t-test*). B, The same experiments were carried on NCI-H460 cells (*** *p*<0.001, *t-test*). Cell viability was measured 72-h post-transfection and was normalized as a percentage of the control and results were expressed as mean ± SEM of three independent experiments. C–D, a cell-cycle distribution of NCI-H23 (C) and NCI-H460 (D) cells transfected with si-Orai3 or si-CTL carried out by flow cytometry of the cells stained with Propidium Iodide. *Inserts*: traces representing distribution of the cells in the cell cycle. Asterisks denote statistical significance as compared to control cells; ^***^ *p*<0.001 of three independent experiments (t-test). E–F, An apoptosis assay carried out by Annexin V staining. Orai3 silencing failed to affect NCI-H23 (E) and NCI-H460 (F) apoptosis.

A 72-h treatment with si-Orai3 significantly reduced the cell number (43±4.1% and 62.5±2.6% for NCI-H23 and NCI-H460 cells respectively, *p*<0.001, Fig. 5A–B). We then examined whether inhibition of Orai3 expression causes NSCLC cell death leading to the reduction in cell number. Down-regulation of Orai3 had no effect on cell death of NCI-H23 or NCI-H460 (Fig. S4 A–B). We have also performed cell counting by Trypan Blue exclusion assay in 48-h after Orai3 silencing and we found a 26% decrease of cell viability in both cell lines without any effect on cell mortality (Fig. S4C–D). These experiments allowed us to conclude that Orai3 plays an important role in NSCLC cell proliferation.

In breast cancer cells, we have previously shown that Orai3 silencing induced cell cycle arrest in the G1 phase [19]. We therefore examined possible alterations in the cell cycle in NSCLC cells with the Orai3-knockdown by flow cytometry. Cells were transfected either by si-Orai3 or siRNA-control for 72-h. Figure 5C–D shows the cell cycle distribution of NCI-H23 and NCI-H460 lung cancer cells. Orai3 silencing led to cell cycle arrest, with cell accumulation in the G0/G1 phase (61.4±2.7% vs 48.8±2.8%, *p*<0.001 for NCI-H23, Fig. 5C, and 68±3.4% *vs* 52±5.1%, *p*<0.001 for NCI-H460, Fig. 5D). At the same time, a decrease in cell number in S and G2/M phases has been observed in both NCI-H23 and NCI-H460 cells transfected with si-Orai3 (Fig. 5C–D). These data demonstrate that Orai3 knockdown causes a cell cycle arrest at G0/G1 phase in NSCLC cells.

It has been reported that Orai3 affects cell survival, and inhibition of Orai3 increases apoptosis [19]. We therefore investigated the effect of Orai3 inhibition on apoptosis using Annexin V, Propidium Iodide double-staining by flow cytometry. Orai3 silencing failed to induce apoptosis in both cell lines (Fig. 5E–F).

### Orai3 regulates Cyclins and cdk expression

**Figure 6:**
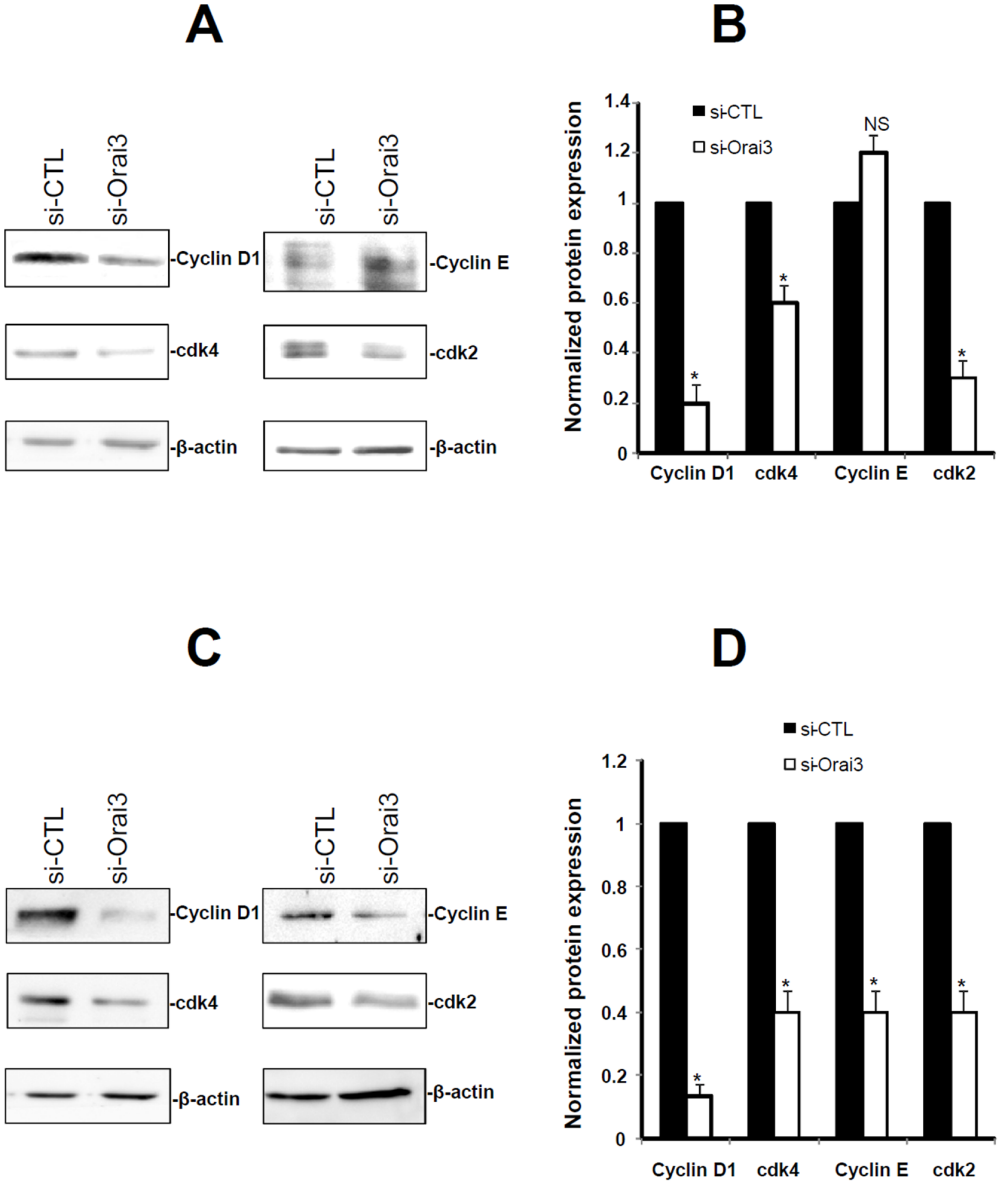
Silencing of Orai3 reduced the up-regulation of cyclin and CDK expression protein levels induced by serum. Cells were transfected by si-Orai3 or si-CTL during 72-h and the expression levels of cell cycle protein were analyzed by Western blotting. A, Representative immunoblots of the expression of cyclin D1, E, Cdk4 and Cdk2 in NCI-H23 cells transfected with si-CTL or si-Orai3. B, Protein levels were quantified and normalized to actin. The indicated values are mean ± SEM of 3 independent experiments, *p<0.05, Mann-Withney test. C, Representative immunoblots of the effect of si-Orai3 on cyclin D1, cyclin E, Cdk4 and Cdk2 expression in NCI-H460 cells. D, Protein levels were quantified and normalized to actin. The indicated values are the mean ± SEM of 3 independent experiments, *p<0.05, Mann-Withney test.

To further clarify the mechanism by which Orai3 knockdown impacts the cell cycle of lung cancer cells, we analyzed the expression of the main cell cycle regulatory proteins by Western blotting. Orai3 silencing decreased the expression of cyclin D1 (49.7±21% for NCI-H23 and 79.7±9.8% for NCI-H460 cells, *p*<0.05, Fig. 6A–B–C–D), Cdk4 (38.7±19% for NCI-H23, 69±13% for NCI-H460 cells, *p*<0.05, Fig. 6A–B–C–D), and Cdk2 (37.4±19% for NCI-H23 and 62.6±13.7% for NCI-H460 cells, *p*<0.05, Fig. 6A–B–C–D). Furthermore, down-regulation of Orai3 decreased cyclin E expression by 49.4±18.5% in NCI-H460 cells, but was without any effect on cyclin E expression in NCI-H23 cells (Fig. 6A–B–C–D). Altogether, these results indicate the involvement of Orai3 in the cell cycle progression and therefore in cell proliferation.

### Orai3 down-regulation inhibited Akt activation

**Figure 7:**
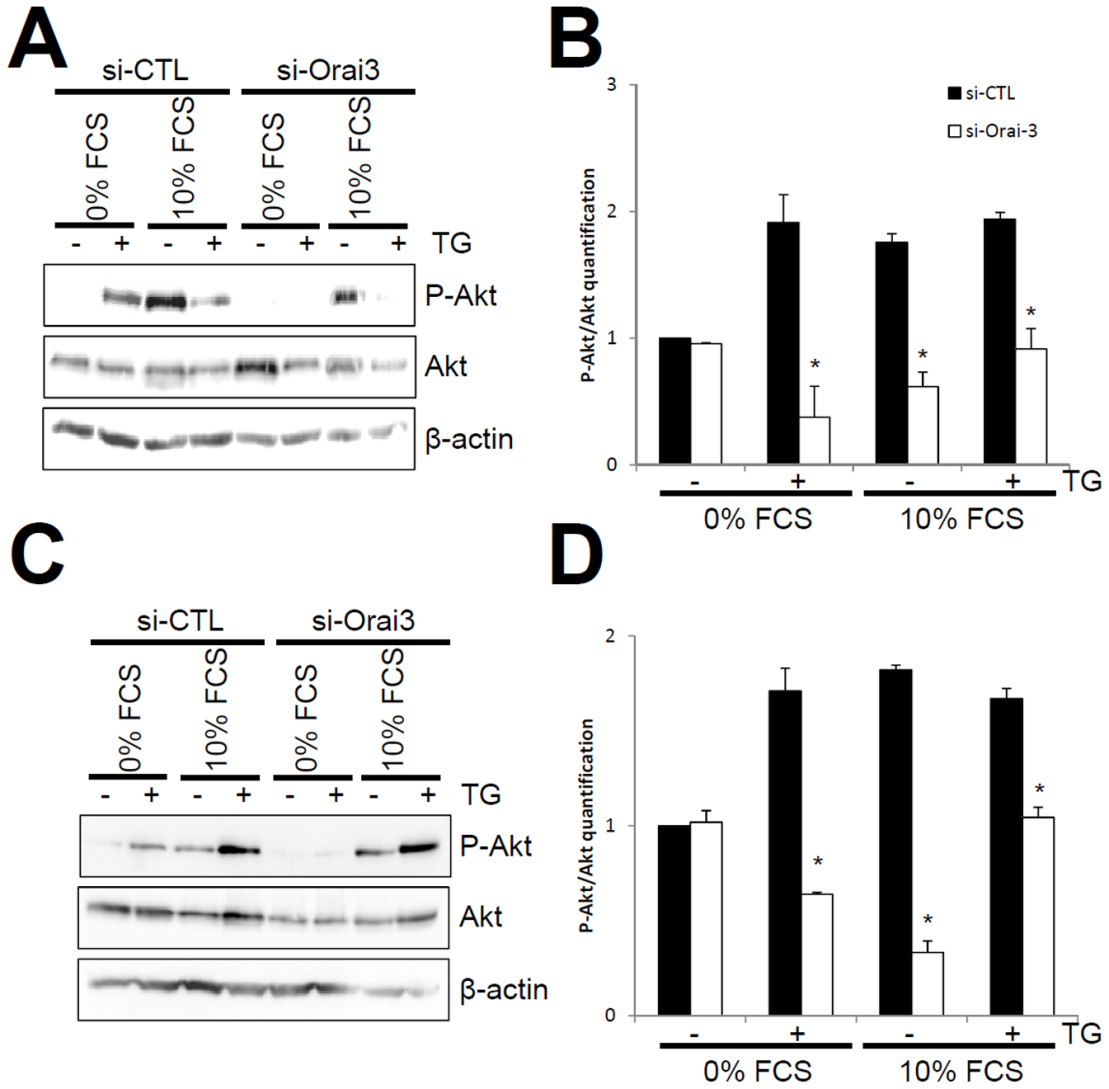
Effect of si-Orai3 on thapsigargin and serum induced AKT phosphorylation. A, B, Representative western blotting of P-Akt and Akt proteins in NCI-H23 (A) and NCI-H460 cells (B) transfected with si-CTL or si-Orai3. Each siRNA was tested in 0% serum (FCS), 0% FCS+1 µM thapsigargin (TG), 10% FCS, and 10% FCS plus 1 µM TG. The quantification of the ratio P-Akt/Akt in NCI-H23 and NCI-H460 cells using densitometric analyses is shown in C and D (n = 2, *p*<0.05, One Way Anova on Ranks).

In order to highlight the mechanisms, by which Orai3 through the SOCE regulates proliferation and cell cycle of non-small cell lung adenocarcinoma, we analyzed, using Western blot, Akt activation when Orai3 is silenced. Indeed, many studies suggest that Akt phosphorylation is responsible for lung cancer cell proliferation [17], [24], [16]. NCI-H23 and NCI-H460 cells transfected with si-CTL or si-Orai3 were starved overnight and then treated for 10 min with 1 µM thapsigargin (TG), serum (FCS, 10%), or both to induce endoplasmic reticulum Ca^2+^ release. Akt activation was analyzed based on the enzyme phorphorylation monitored with anti-phospho-Akt antibody. In both NCI-H23 and NCI-H460 transfected with si-CTL, Akt was activated with TG in 0% FCS (91.3±22.1% for NCI-H23 and 71.03±11.9% for NCI-H460, *p*<0.05, Fig. 7A–B–C–D), or with 10% FCS (75.6±6.89% for NCI-H23 and 82.1±2.55% for NCI-H460, *p*<0.05, Fig. 7A–B–C–D). Silencing of Orai3 decreased Akt phosphorylation triggered by TG in 0% FCS (80.3±24.5% and 62.6±0.9%), 10% FCS alone (64.7±11.41% and 81.7±6.1%), or by both TG and serum (52.8±16.1% and 37.4±5.3%) in NCI-H23 (Fig. 7A–C) and NCI-H460 cells (*p*<0.05, Fig. 7B–D). These results suggest that Ca^2+^ entry via Orai3 is able to activate Akt pathway in NSCLC cell lines.

## Discussion

Our results show that NCI-H23 and NCI-H460 cells express Orai1, Orai2, Stim1 and Stim2. SOCE is inhibited by low concentrations of lanthanides (5 µM Gd^3+^), but neither Orai1, nor Orai2 regulates it in the NSCLC cells. Interestingly, SOCE is increased by 2-APB application and decreased by silencing of Orai3. Importantly, we found that Orai3 contributes to non-small cell lung adenocarcinoma cell proliferation and cell cycle progression likely through Akt pathway. Moreover, Orai3 is overexpressed in tumoral lung tissues compared to normal ones, and correlates with high tumor grade. Since its role in proliferation and its increased expression in NSCLC samples, Orai3 may thus be a potential target for adenocarcinoma lung therapy.

Store-operated Ca^2+^ entry mediated by Stim/Orai is involved in the control of several cellular functions, including cell growth and proliferation. Indeed, Orai1, STIM2, but not STIM1 regulate cell cycle progression and proliferation in HEK293 cells [15]. Recently, Motiani et al. [18] have reported the expression of Orai and STIM in five breast cancer cell lines and demonstrated that Orai3 is the major component in the native store-operated channels in ER positive breast cancer cell lines [18]. Furthermore, we have reported that Orai3 knockdown inhibits proliferation of MCF-7 and T-47D human breast cancer cells [19]. Consistent with these studies, the current work provides new evidence that Orai3 is crucial for SOCE and cell proliferation in lung cancer cell lines. In lung adenocarcinoma, predominant studies have been performed on one lung cell line, A-549, where TRPC1 and Orai1 have been reported to contribute to SOCE [16], [17]. Thus, there is a crucial need to characterize SOC partners in other NSCLC cell lines. Our data demonstrate that SOCE is sensitive to Gd^3+^ in both NCI-H23 and NCI-H460 cells. Moreover, Orai (1–3) and Stim (1–2) are expressed in these cell lines. Silencing of the Orai1 or Orai2 failed to affect SOCE, while Orai3 knockdown significantly reduced both basal Ca^2+^-fluorescence and SOCE. Our results are in support of Orai3 as the main SOCE pathway in NSCLC cell lines (NCI-H23 and NCI-H460) that participates in the regulation of intracellular Ca^2+^ concentration.

Cell proliferation is finely regulated by the progression through three distinctive phases of the cell cycle (G0/G1, S, and G2/M); thus, cell cycle arrest is considered one of most common causes of the inhibition of cell proliferation. We have previously shown in breast cancer cells that Orai3 silencing suppressed cell proliferation, arrested cell cycle in the G0/G1 phase, and this phenomenon is associated with a reduction in CDKs 4/2 and cyclins D1 and E expression. Here, we show that Orai3 controls NSCLC cell growth via regulating G1 to S phase cell cycle transition. Indeed, cyclin D1 and E are essential regulators of G1 and G1/S transition. Our results show that the expression of cyclin D1 and cyclin E, as well as the expression of CDK4/2 were regulated by Orai3. These findings in different types of tumors indicate that Orai3 is likely a key regulator of Ca^2+^-mediated cell cycle progression. In addition, Orai-3 silencing did not induce apoptotic cell death. This result is however different from its anti-apoptotic effect observed in breast cancer [19].

Ca^2+^ signaling is required for progression through G1, the G1/S transition and G2/M in several cell types [25], [26], [27]. Ca^2+^ influx activates Ca^2+^-dependent transcription factors leading to the expression of cell cycle regulatory proteins, such as cyclins, CDKs, and to the inhibition of CDKi expression [28], [29], [19]. In agreement with these considerations, it has been reported that calcium entry through TRPV6 channel induces a subsequent downstream activation of Nuclear Factor Activated T cell (NFAT) leading to LNCaP prostate cell proliferation [30]. Moreover, in human hepatoma cell line Huh-7, it has been also shown that Ca^2+^ entry involving TRPC6, together with STIM1 and Orai1, increases cyclin D1 expression. Furthermore, we have also reported that Orai3 knockdown accumulates the cells in G1 phase and decreases expression of cyclins D1 and E [19]. In this context, we suggest that Ca^2+^ entry through Orai3 may control cell cycle progression by regulating expression of cyclins and CDKs in NCI-H23 and NCI-H460 lung cancer cells.

The PI-3K/Akt signaling pathway is central to cell proliferation and survival and is constitutively activated in lung cancer cells [31]. Indeed, blockade of PI-3K/Akt pathway strongly blocks cell cycle along with a decreased expression of cyclins D1 and D3 [17]. Moreover, reducing Ca^2+^ entry, via SOCE, inhibits Akt phosphorylation and reduces EGF-induced proliferation in A-549 cells [16]. In the present study, we found that Orai3 silencing suppressed Akt phosphorylation in both cell lines suggesting that the proliferative effect of Orai3 is, in part, attributed to the Akt signaling pathway in lung cancer cells.

Finally, our results clearly show that Orai3 channels are expressed in both normal and tumor lung tissues. However, Orai3 is over-expressed in tissues from 60 patients presenting non small cell lung adenocarcinoma versus non tumoral adjacent tumoral tissues. Moreover, the expression of Orai3 increases in high tumor grade. This result suggests the involvement of Orai3 in NSCLC development.

In conclusion, our study highlights Orai3 as a major regulator of cell cycle progression and therefore growth of NSCLC cells and makes it as an interesting potential bio-marker and/or target for adenocarcinoma lung therapy.

## Supporting Information

Figure S1 **Orai-1 and Orai-2 mRNA determined in NCI-H23 (A) and NCI-H460 (B) cells transfected with si-Orai1 (i), si-Orai2 (ii), or si-CTL using Q-PCR.** Si-Orai1, as well as si-Orai2 decreased the Orai1 or Orai2 transcript levels normalized to β-actin in NCI-H23 (A) and NCI-H460 (B) (n = 2, *p<0.05, **p<0.01, ***p<0.001, Mann-Withney test).
(TIF)

Figure S2 **Orai3 mRNA (i) and protein (ii) levels in NCI-H23 (A) and NCI-H460 (B) cells transfected with si-CTL and si-Orai3, as determined by Q-PCR and Western blot.** Si-Orai3 decreased Orai3 transcript and protein levels normalized to β-actin in NCI-H23 (A) and NCI-H460 (B) (n = 3, *p*<0.001, Mann-Withney test). Orai-1 mRNA (i) and protein (ii) levels detected by Q-PCR and Western blot in NCI-H23 (C) and NCI-H460 (D) cells transfected with si-CTL and si-Orai3 (n = 2, Mann-Withney test).
(TIF)

Figure S3 **Orai3 channel is functional in NCI-H23 cells and contributes to the basal calcium entry (A).** Slope values are: for si-CTL: −0.52±0.2 (n = 69, 64.4%) and for si-Orai3: −0.41±0.4 (n = 53, 67.9%). B: Orai3 channel is functional in NCI-H460 cells and contributes to the basal calcium entry. Slope values are: for si-CTL: −0.46±0.2 (n = 92, 78.6%) and for si-Orai3: −0.39±0.3 (n = 85, 61.6%).
(TIF)

Figure S4 **Down-regulation of Orai3 had no effect on cell death of NCI-H23 (A) or NCI-H460 (B).** Cells were transfected with si-CTL and si-Orai3. A 24-h treatment with si-Orai3 was without effect on cell viability in both NCI-H23 (C) and NCI-H460 (D), while after 48-h treatment, si-Orai3 significantly reduced the cell number for NCI-H23 (C) and NCI-H460 cells (D). Histogram is representative of three independent experiments and results are expressed as means ± S.D (t-test).
(TIF)
