# Correction: Orai3 Constitutes a Native Store-Operated Calcium Entry That Regulates Non Small Cell Lung Adenocarcinoma Cell Proliferation

**DOI:** 10.1371/journal.pone.0124201

**Published:** 2015-04-01

**Authors:** The PLOS ONE Staff

The second author's name is spelled incorrectly. The correct name is: Nazim Benzerdjeb. The correct citation is: Ay A-S, Benzerdjeb N, Sevestre H, Ahidouch A, Ouadid-Ahidouch H (2013) Orai3 Constitutes a Native Store-Operated Calcium Entry That Regulates Non Small Cell Lung Adenocarcinoma Cell Proliferation. PLoS ONE 8(9): e72889. doi:10.1371/journal.pone.0072889
