# Supplementary figures and images for: Orai3 Constitutes a Native Store-Operated Calcium Entry That Regulates Non Small Cell Lung Adenocarcinoma Cell Proliferation
Source: PLoS One. 2013 Sep 13;8(9):e72889. doi: 10.1371/journal.pone.0072889 (PMC3772818; doi:10.1371/journal.pone.0072889)

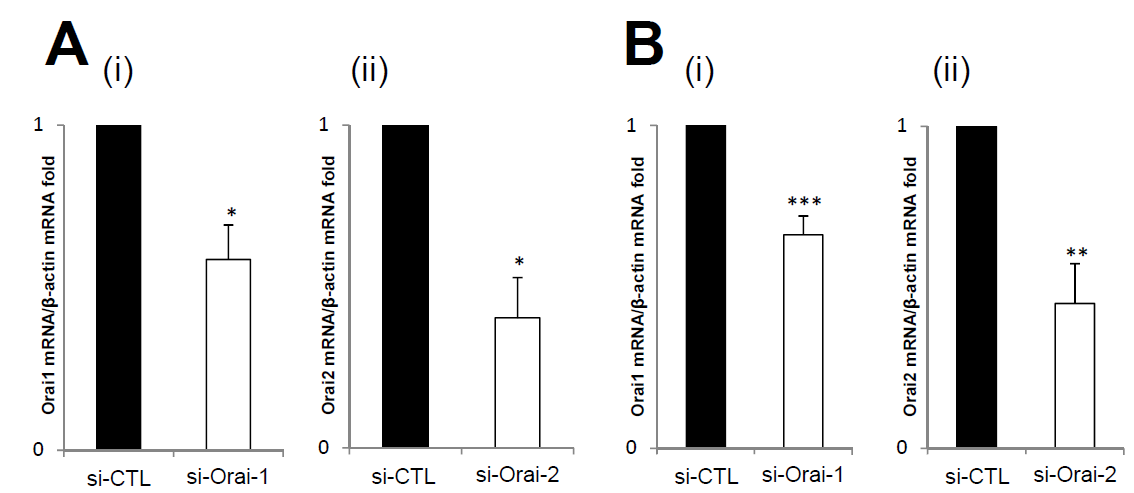

Supplement: Figure S1 — Orai-1 and Orai-2 mRNA determined in NCI-H23 (A) and NCI-H460 (B) cells transfected with si-Orai1 (i), si-Orai2 (ii), or si-CTL using Q-PCR. Si-Orai1, as well as si-Orai2 decreased the Orai1 or Orai2 transcript levels normalized to β-actin in NCI-H23 (A) and NCI-H460 (B) (n = 2, *p<0.05, **p<0.01, ***p<0.001, Mann-Withney test). (TIF) [file pone.0072889.s001.tif]

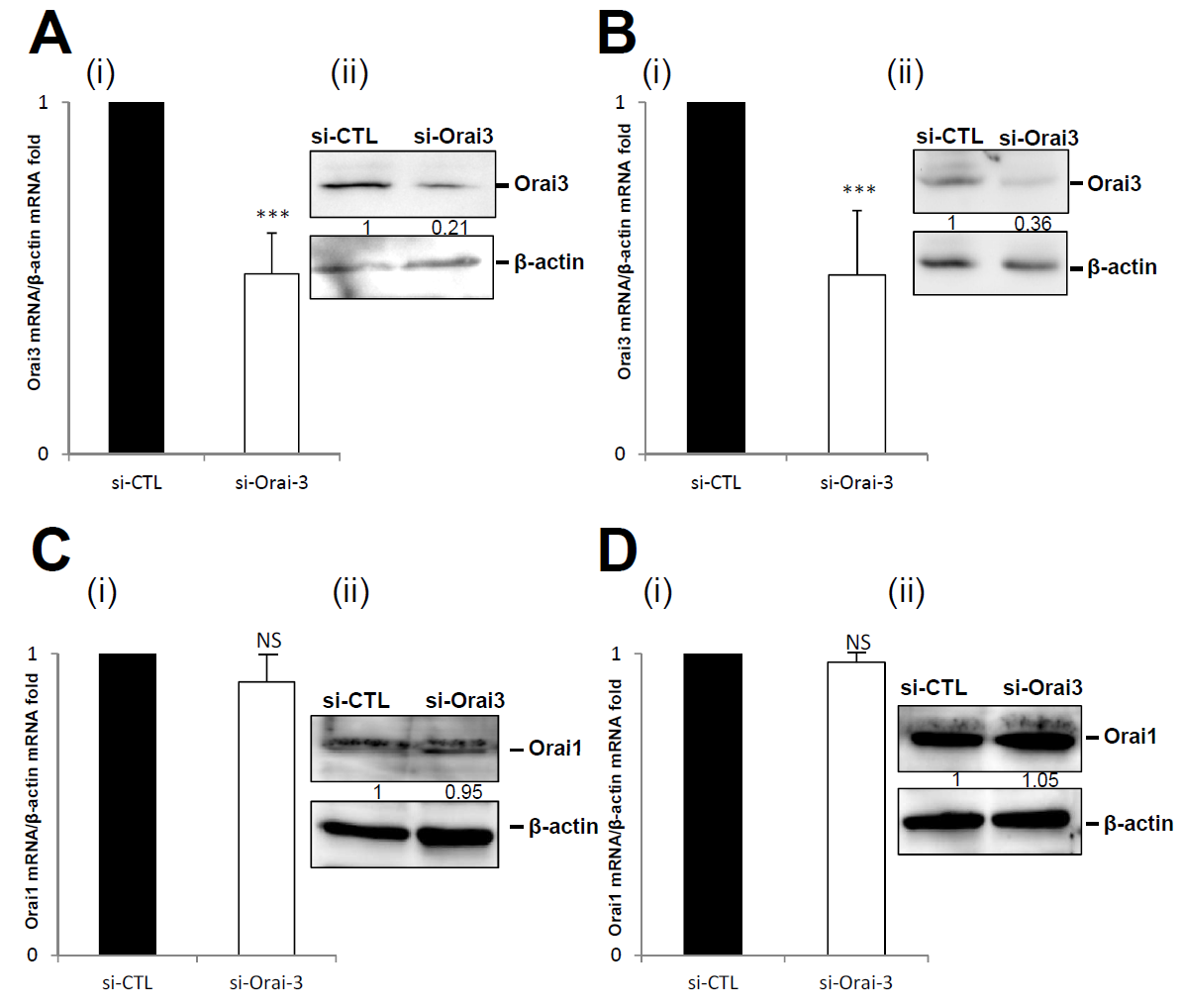

Supplement: Figure S2 — Orai3 mRNA (i) and protein (ii) levels in NCI-H23 (A) and NCI-H460 (B) cells transfected with si-CTL and si-Orai3, as determined by Q-PCR and Western blot. Si-Orai3 decreased Orai3 transcript and protein levels normalized to β-actin in NCI-H23 (A) and NCI-H460 (B) (n = 3, p<0.001, Mann-Withney test). Orai-1 mRNA (i) and protein (ii) levels detected by Q-PCR and Western blot in NCI-H23 (C) and NCI-H460 (D) cells transfected with si-CTL and si-Orai3 (n = 2, Mann-Withney test). (TIF) [file pone.0072889.s002.tif]

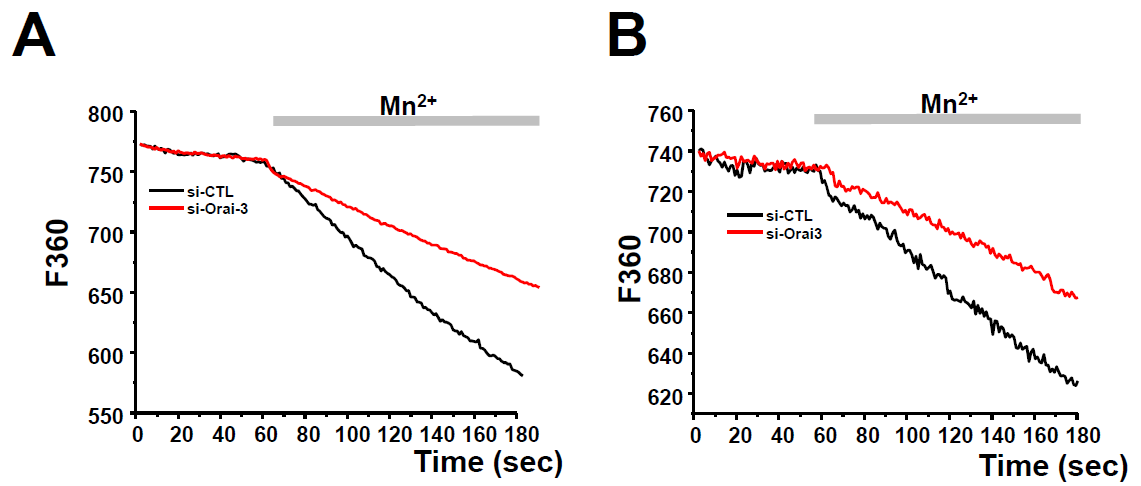

Supplement: Figure S3 — Orai3 channel is functional in NCI-H23 cells and contributes to the basal calcium entry (A). Slope values are: for si-CTL: −0.52±0.2 (n = 69, 64.4%) and for si-Orai3: −0.41±0.4 (n = 53, 67.9%). B: Orai3 channel is functional in NCI-H460 cells and contributes to the basal calcium entry. Slope values are: for si-CTL: −0.46±0.2 (n = 92, 78.6%) and for si-Orai3: −0.39±0.3 (n = 85, 61.6%). (TIF) [file pone.0072889.s003.tif]

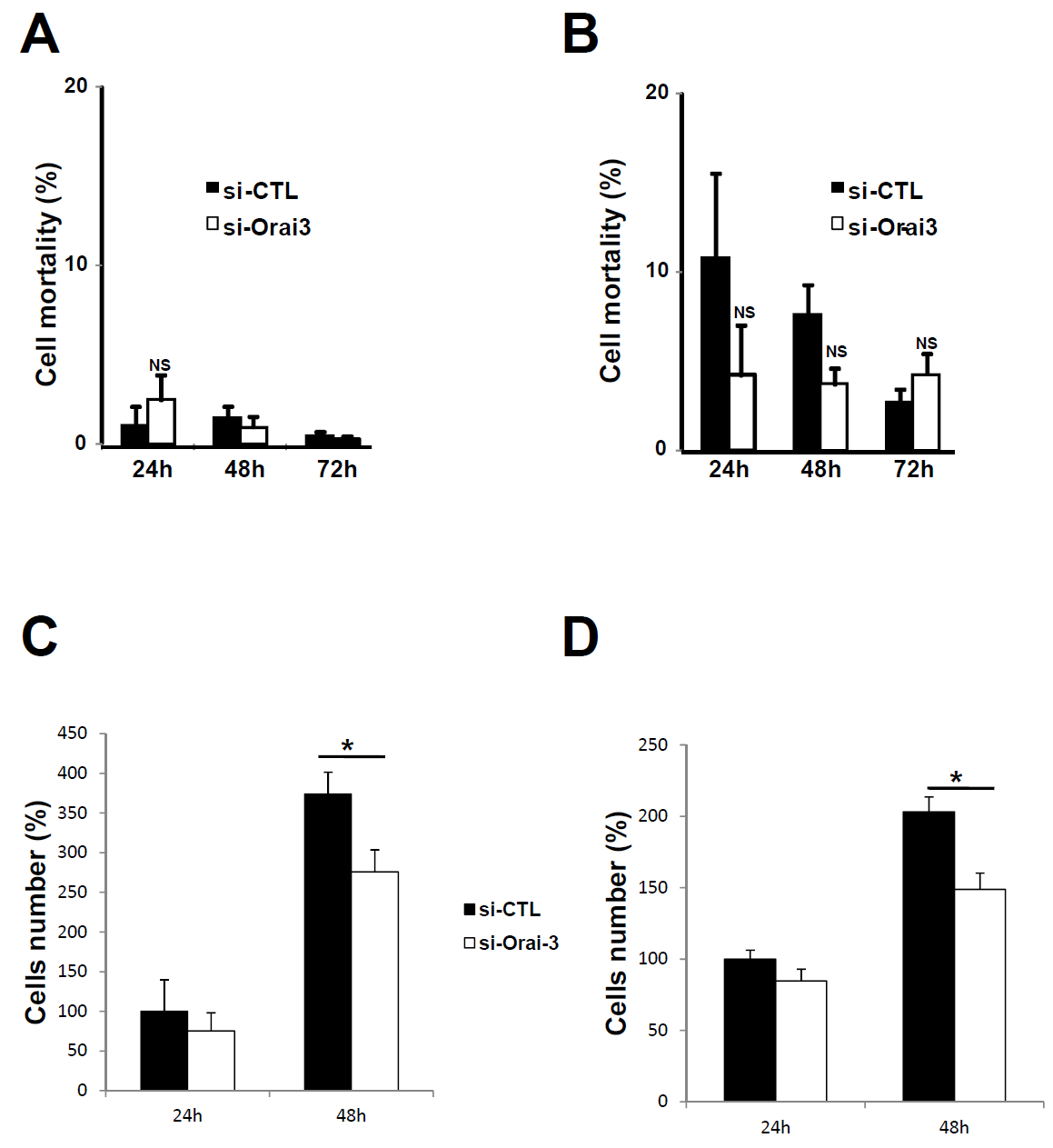

Supplement: Figure S4 — Down-regulation of Orai3 had no effect on cell death of NCI-H23 (A) or NCI-H460 (B). Cells were transfected with si-CTL and si-Orai3. A 24-h treatment with si-Orai3 was without effect on cell viability in both NCI-H23 (C) and NCI-H460 (D), while after 48-h treatment, si-Orai3 significantly reduced the cell number for NCI-H23 (C) and NCI-H460 cells (D). Histogram is representative of three independent experiments and results are expressed as means ± S.D (t-test). (TIF) [file pone.0072889.s004.tif]
